# Lapatinib dysregulates HER2 signaling and impairs the viability of human uveal melanoma cells

**DOI:** 10.7150/jca.88446

**Published:** 2023-10-16

**Authors:** Wenying Shu, Janney Z Wang, Xue Zhu, Ke Wang, Svetlana Cherepanoff, R. Max Conway, Michele C Madigan, Li-Anne Lim, Hong Zhu, Ling Zhu, Michael Murray, Fanfan Zhou

**Affiliations:** 1 The University of Sydney, Sydney Pharmacy School, Faculty of Medicine and Health NSW 2006, Australia.; 2 Department of Pharmacy, Affiliated Cancer Hospital & Institute of Guangzhou Medical University, Guangdong Province 511400, China.; 3 Key Laboratory of Nuclear Medicine, Ministry of Health, Jiangsu Key Laboratory of Molecular Nuclear Medicine, Jiangsu Institute of Nuclear Medicine, Wuxi, Jiangsu Province 214063, China.; 4 SydPath, Department of Anatomical Pathology, St Vincent's Hospital, Darlinghurst, NSW 2010, Australia.; 5 Ocular Oncology Unit, Sydney Eye Hospital and The Kinghorn Cancer Centre, Sydney, NSW 2006, Australia.; 6 Save Sight Institute, The University of Sydney, Sydney, NSW 2006, Australia.; 7 School of Optometry and Vision Sciences, University of New South Wales, Sydney, NSW 2006, Australia.; 8 Zhejiang Province Key Laboratory of Anti-Cancer Drug Research, College of Pharmaceutical Sciences, Zhejiang University, Hangzhou, Zhejiang Province 310058, China.

**Keywords:** uveal melanoma, lapatinib, anti-cancer, anti-metastatic, HER2 inhibition

## Abstract

Uveal melanoma (UM) is the principal type of intraocular malignancy in adults. Up to 50% of UM patients develop metastatic disease with very poor survival. There are few drugs available to treat the primary or metastatic UM. This study was undertaken to evaluate the anti-cancer effect of lapatinib and corroborate the potential of HER2 inhibition in the treatment of UM.

The anti-UM activity of lapatinib was assessed using cell viability, cell death and cell cycle analysis, and its anti-metastatic actions were evaluated using would healing, invasion and colony formation assays. Immunoblotting was used to substantiate the actions of lapatinib on apoptotic and HER2 signaling. The anti-UM activity of lapatinib was further evaluated in a UM xenograft mouse model.

Lapatinib decreased the viability of four UM cell lines (IC_50_: 3.67-6.53 µM). The antiproliferative activity of lapatinib was corroborated in three primary cell lines isolated from UM patient tumors. In UM cell lines, lapatinib promoted apoptosis and cell cycle arrest, and strongly inhibited cell migration, invasion and reproductive cell growth. Lapatinib dysregulated HER2-AKT/ERK/PI3K signalling leading to the altered expression of apoptotic factors and cell cycle mediators in UM cell lines. Importantly, lapatinib suppressed tumourigenesis in mice carrying UM cell xenografts.

Together the present findings are consistent with the assertion that HER2 is a viable therapeutic target in UM. Lapatinib is active in primary and metastatic UM as a clinically approved HER2 inhibitor. The activity of lapatinib in UM patients could be evaluated in future clinical trials.

## Introduction

Uveal melanoma (UM) is a rare cancer that is very different from its cutaneous counterpart 1. As the primary intraocular malignancy, UM accounts for over 85% of ocular tumours and is also the second most common type of melanoma (~5% of all cases) 2, 3. The incidence of UM is similar in males and females and affects both eyes equally; however, it is more frequently identified in Caucasians and in adults aged over 40 3, 4.

Although the survival rate is around 84% for early-stage UM (AJCC stages I and II), patients often experience treatment delays due to the difficulty in distinguishing tumours from benign tissues. UM tumours are often asymptomatic until they reach a significant size 5-7. Not surprisingly, the mortality rate increases dramatically in late-stage UM 2. Up to 50% of patients develop metastases, particularly in the liver, prior to diagnosis 8, 9. The median survival rate of UM patients with liver metastases is 4-6 months and those whose disease is more advanced may survive <3 months 10-12.

Clinically, enucleation has been widely used in the treatment of primary UM tumours, but this may lead to irreversible eye damage. More recently, other treatment options such as brachytherapy, proton beam therapy and phototherapy, have been used to treat primary UM tumours with the aim of preserving vision 1, 13, 14. Although the current clinical guidelines state that laser- and radio-therapy are the primary treatment for UM primary tumours, the effectiveness of these regimen in patients is limited to early diagnoses and small sized tumours. Nevertheless, such non-pharmacological approaches are unable to prevent metastatic lesions developing in distant tissues. Even though hepatic chemoembolization, isolated hepatic perfusion, intra-arterial chemotherapy, radiotherapy, and surgical resection are effective in the treatment of patients with liver cancer, these approaches are generally ineffective in UM tumours that have metastasized to the liver 12, 15, 16. Therefore, the identification of drugs that can effectively treat primary tumours and prevent metastasis in UM patients would be highly significant.

Both cutaneous melanoma and UM are derived from melanocytes. However, unlike cutaneous melanoma, environmental factors, such as UV radiation and latitude, are not associated with the development of UM. Furthermore, cutaneous melanoma is characterised by mutations that activate the v-raf murine sarcoma viral oncogene homolog B1 (BRAF), neuroblastoma RAS viral oncogene homolog (NRAS), tyrosine-protein kinase (KIT) and phosphatidyinositol 3-Kinase (PI3K) pathways, whereas UM is unrelated to these mutations. UM is characterised by low tumour mutational burden compared to cutaneous melanoma where mutagenic effect of UV light is apparent 17, 18. However, UM displays a distinct genetic profile that may be associated with its development and prognosis 19. Notably, the most common mutations in UM occur in the tumour suppressor gene and the guanine nucleotide binding protein Gαq/Gα11 (GNAQ/11) gene, which accounts for over 40% of genetic mutations in UM, followed by BRCA1 associated protein 1 (BAP1). Mutations within these two regulatory genes result in increased cell growth, proliferation and metastasis leading to poor prognosis in UM 20, 21. Other molecular changes including monosomy of chromosome 3, amplification or gain of chromosome 8q all appear to contribute to the grim prognosis of UM patients 22, 23. Drugs that are used clinically for the treatment of cutaneous melanoma are ineffective in the treatment of UM 1, 24-29.

The epithelial growth factor receptor (EGFR) family consists of 4 members: ErbB1-4. ErbB receptors are transmembrane proteins that have a cytoplasmic binding domain, a transmembrane domain and an intracellular domain that interacts with downstream signalling pathways. Receptor activation causes hetero- or homo-dimerization, followed by autophosphorylation on tyrosine residues in the intracellular kinase domain. Activation of downstream pathways, such as the PI3K/protein kinase B (AKT), Ras/mitogen-activated protein kinase (MEK)/extracellular-signal-regulated kinase (ERK), phospholipase C Gamma (PLCγ)/protein kinase C (PKC), and janus kinase (JAK)/signal transducer and activator of transcription (STAT) cascades, regulates cell survival, proliferation, differentiation, motility, apoptosis, survival, invasion, migration, adhesion, and angiogenesis 30, 31. Therefore, ErbB isoforms have been widely studied as cancer drug targets 32. Among the 4 ErbBs, HER2 has an established role in breast cancer. It has also been shown to have an important role in the prognosis of various other cancers such as gastric, biliary tract, colorectal and non-small cell lung cancer. The overexpression/amplification of HER2 in these cancers may contribute to the poor prognosis and more aggressive tumours 33. Hence, it is important that the role of HER2 is evaluated in UM.

With advances in gene profiling, multi-kinase inhibitors have been suggested to have potential value in the development of new treatment strategies in UM 34-36. We recently tested several multi-kinase inhibitors in UM cell lines 37. We found that afatinib, which is a potent inhibitor of multiple ErbB receptors, including EGFR, HER2 and HER4, induced cell death and prevented cell migration in UM cell lines. It is noteworthy that EGFR and HER4 are not commonly expressed in UM tumours 25, 34-41. Therefore, neither of these receptors is likely to be a primary molecular target for UM drug development. The finding that afatinib dysregulated HER2 signaling to exert its anti-UM activity suggests that HER2 could be a novel therapeutic target in UM 37. To substantiate the potential clinical significance of HER2 in UM, we investigated the anti-UM activity of the HER2 inhibitor lapatinib in the present study. Lapatinib was selected because it is clinically approved for use in patients with HER2-positive breast cancers that are resistant to the front-line agent trastuzumab 42. Thus, lapatinib has the potential advantage that it may be more rapidly translated to clinical application in UM.

Indeed, we have demonstrated some of specific applications of Lapatinib on UM in Research Square, which is not a recognized publisher. The purpose of the preprint to receive comments from peers in the field that could further improve the study for subsequent formal publication by an appropriate journal. Indeed, we have improved the present study by including additional data based on the feedback we received on the preprint (eg. Fig 6E).

## Material and Methods

### Reagents and biochemicals

Dulbecco's Modified Eagle Medium (DMEM), Fetal Bovine Serum (FBS), Insulin-Transferrin-Selenium (ITS), L-Glutamine, Penicillin-Streptomycin (P/S) and Roswell Park Memorial Institute Medium (RPMI-1640) were purchased from Thermo Scientific (Lidcombe, NSW, Australia). Giant cell tumour (GCT) conditioned medium was obtained from United Biosciences (Carindale, QLD, Australia). The β-actin antibody, dimethyl sulfoxide (DMSO) and thiazolyl blue tetrazolium bromide (MTT) were purchased from Sigma-Aldrich (Castle Hill, NSW, Australia). Lapatinib was from Selleck Chemicals (Houston, Texas, USA), dissolved in DMSO and stored at -20ºC. Antibodies were purchased from Cell Signalling Technology (Danvers, MA, USA): Akt (pan, Cat. #: 4685), Bcl2-associated X protein (Bax; D2E11, Cat. #: 5023), Bcl-XL (54H6, Cat. #: 2764), cyclin D1 (Cat. #: 55506), GAPDH (D16H11, Cat. #: 5174), HER2/ErbB2 (Cat. #: 4290), ERK (Erk, Cat. #: 4695), PI3K p85 (19H8, Cat. #: 4257), phospho-Akt (Ser473, Cat. #: 4060), phospho-HER2/ErbB2 (Tyr1196, Cat. #: 6942), phospho-PI3K p85 (Tyr458)/p55 (Tyr199) (E3U1H, Cat. #: 17366), phospho-ERK (Thr202/Tyr204, Cat. #: 4370) and STAT1 (D1K9Y, Cat. #: 14994). The FITC Annexin V Apoptosis Detection Kit II was purchased from BD Bioscience (North Ryde, NSW, Australia). Goat anti-mouse and anti-rabbit IgGs that were conjugated with horseradish peroxidase were obtained from Bio-strategy delivery technology (Tullamarine, VIC, Australia). PVDF membranes were from Merck Millipore (Bayswater, VIC, Australia).

### UM cell lines

UM cell lines used in this study were obtained as indicated previously 37. All cell lines were regularly checked for mycoplasma with MycoAlert Mycoplasma Detection kit (Lonza, Mount Waverley, VIC Australia) to ensure optimal viability. RPMI-1640 was used to culture C918, Mel202, MP46 and 92.1 cells and DMEM was used to maintain OMM-1 and OCM-1 cells. All culture media was supplemented with 10% heat-inactivated FBS (v/v), 1% L-Glutamine and 1% P/S (Thermo Scientific, Lidcombe, NSW, Australia). Cells were incubated in a humidified incubator (5% CO_2_) at 37 °C.

The early literature indicated that the C918 cell line was derived from primary UM 43. According to the previous report 44, the STR profile of the C918 cell line seemed identical to that of the cutaneous melanoma C8161 cell line 45. However, the reference to the STR profile of C8161 cells could not be verified - either on the journal website or through other resources eg. Pubmed, Medline. Therefore, the authenticity of the information contained within Yu et al.'s paper has still yet to be fully substantiated. We compared the STR profiles of the C918 cell line obtained from the Swiss Institute of Bioinformatics and the C8161 cell line provided by Lonza. We found that these two STR profiles were quite different. In addition, we undertook a proteomics analysis to compare C918 cells with the malignant human melanoma cell line A375. We found that that p75NTR and S100 were down-regulated in C918 while MITF was up-regulated. This is consistent with a previous report that immunophenotyping for proteins such as HMB-45, HMB-50, p75NTR, S100 and MITF could be used to distinguish uveal and cutaneous melanomas 46. Based on the evidence from genotyping and immunophenotyping studies described above, we consider that C918 and C8161 appear to be different cell lines. Thus, our study is consistent with a number of other recent reports that C918 is an in vitro model of UM 47-49.

### Cell viability assay

Assays of MTT reduction were used to determine cell viability after lapatinib treatment. UM cells were cultured in 96 well plates (2x10^4^cell/well). Cells were treated with various concentrations of lapatinib in RPMI-1640 or DMEM containing 1% FBS. MTT (0.5 mg/mL) was added 24 h later and, after incubation in the dark for 2 h, cells were washed with phosphate-buffered saline (PBS; 0.154 M NaCl, 0.001 M KH_2_PO_4_, 0.003 M Na_2_HPO_4_; pH 7.4), DMSO was added, and plates were shaken for 10 min at room temperature. Absorbance was measured at 550 nm in a microplate reader (Model 680, Bio-Rad, Gladesville, NSW, Australia). IC_50_ values were calculated by non-linear regression of MTT inhibition as a function of drug concentration (GraphPad Prism 7.0; San Diego, CA).

### Annexin V/propidium iodide flow cytometry assay

UM cells were seeded and treated with lapatinib (5 µM) in medium containing 1% FBS. Cells were collected 24 h after treatment, washed with PBS, suspended, and stained with annexin V and propidium iodide (PI) for 20 min at room temperature. Samples were subjected to flow cytometry (Guava easycyte; Merck Millipore, Bayswater, VIC, Australia) and apoptotic and necrotic cells were quantified as described previously 37.

### Cell cycle analysis

Cells were seeded and treated with lapatinib (5 µM) for 24 h, then harvested and washed twice in PBS before fixing overnight in cold 70% ethanol at -20 °C. The ethanol was removed, samples were washed with PBS and then stained in the dark with PI for 30 min at 37 °C, after which they were analysed by flow cytometry (Guava easycyte).

### Scratch-wound cell migration assays

Cells were cultured on 24-well microplates (5 × 10^4^ cells/well). After 24 h scratches were made with a Wound Maker instrument (Sartorius, Dandenong South, VIC, Australia). Cells were washed with PBS and incubated in medium containing 1% FBS (v/v) and lapatinib (5 µM) for 24 h. Cells were incubated at 37°C and photos were taken at 2 h intervals with an Essen IncuCyte S3 instrument (10X magnification; Sartorius). Cell migration rates were determined using Image J software (National Institutes of Health, USA). Migration rate was calculated as:



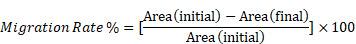



Area (initial) is the area of the scratch measured immediately after wounding (t = 0 h).

Area (final) is the area of the wound measured 24 h after the scratch was applied.

### Matrigel invasion assay

UM cells were seeded into 96-well plates (3-4 × 10^4^/well). After 24 h, scratches were made as described above. Cell debris was removed by washing with PBS. MatriGel Matrix (BD Falcon, Chatswood, NSW, Australia) diluted in culture medium (200 - 800 µg/mL) was added to each well and allowed to solidify for 1 h at 37 °C. Following this, cells were treated with lapatinib (5 µM) and treatments were replenished at 24h intervals. The plates were imaged over 24 to 72 h with an Essen IncuCyte S3 instrument. Cell invasion rates were determined using Image J software and the invasion rate was calculated as:



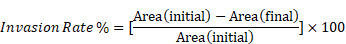



### Colony formation assay

Cells were treated with lapatinib (5 µM) in 12 well plates and then aliquoted into 24-well plates (200 cells/well) for 6-8 days. Methanol (100%) was used to fix cells before staining with crystal violet. Colony growth was defined microscopically as a cluster of at least 50 cells. Photos were taken in an Essen IncuCyte S3, using whole-well scan mode at 4 X magnification. Image J software was used to identify the leading edge of the cell population.

### Western blot

Cells were treated with lapatinib and incubated for 24 h before they were harvested with lysis buffer containing NP-40 (1% IGEPAL, 150 mM NaCl and 50 mM Tris, pH 7.8) containing protease inhibitors. Lysates were then centrifuged at 15,000 rpm (10 min, 4 °C) to separate protein-containing supernatants and cell remnants; supernatant fractions were denatured on a heat block.

Proteins in supernatant fractions were separated by electrophoresis, transferred to a PVDF membrane and incubated in 5% non-fat milk dissolved in PBS containing Triton 0.05% X-100 (PBST) at room temperature for 30 min. The membranes were cut prior to hybridisation with different antibodies. The membranes were incubated overnight with a primary antibody at 4 °C. Membranes were washed three times with PBST and were then incubated at room temperature with a secondary antibody for 1h. Signals were detected using chemiluminescence (SuperSignal West Pico, Thermo Scientific, Lidcombe, NSW, Australia) and were visualized with ImageQuant LAS500 (GE Health Care, Silverwater, NSW, Australia) or Chemidoc image machine (Bio-Rad, Gladesville, NSW, Australia).

### Primary UM tumour derived cell lines

Human UM tumour samples were obtained with approval from St. Vincent's Hospital Sydney Human Ethics Committee (HREC/17/SVH/346) and experiments were strictly conducted as per the relevant guidelines and regulations. All the informed consent for the patient samples used in this study have been obtained. Tumour tissues were surgically removed, cut into segments, treated with trypsin-EDTA and then washed three times with PBS (pH 7.4). Individual cells were collected and incubated at 37°C in RPMI-1640 medium containing 20% FBS (v/v), 1% L-glutamine, 1% P/S, 1% ITS and 2% GCT under a 5% CO_2_ atmosphere. All experiments were conducted in cells at passage 2 to 5.

### UM xenograft mouse model

Animal ethics approval was obtained from the Laboratory Animal Ethics Committee of Jiangsu Institute of Nuclear Medicine (Wuxi, China).

Animal experiments were conducted in accordance with approved protocols and regulations. The study Results were reported according to ARRIVE guidelines 50. C918 cells were mixed in a 2:1 ratio with Matrigel and injected subcutaneously in BALB/c nude mice (5 weeks old; male; Chang Zhou Cavens Laboratory Animal Co., Ltd, Changzhou, China). Tumour volume was measured with callipers every 3 d until they reached ~100 mm^3^ in size. Tumour volumes were calculated as (a × b^2^)/2, where a and b are the length and width of the tumours, respectively. Once tumours reached the desired volume (around day 10), mice were randomly assigned to two groups to receive either lapatinib (25 mg/kg; n=7) or vehicle (n=7) once daily by intraperitoneal injection. Body weights and tumour sizes were measured every 2 days for 14 days. Drug administration was continued for 24 days. When treatments were complete, the mice were anesthetised with pentobarbital sodium (50 mg/kg) by intraperitoneal injection. Tumours were excised, weighed, photographed, and fixed in 4% paraformaldehyde for subsequent analysis.

### Positron emission tomography (PET) scanning

^68^Ga Activity was eluted from a ^68^Ge/^68^Ga generator and used to prepare [^68^Ga] Ga-NOTA-PRGD2 tracer, as described previously 25. On the day of scanning, the mice received ~3.7MBq of ^68^Ga NOTA-PRGD2 under anaesthesia via tail vein injection. Dynamic imaging acquisition was conducted for 60 min after tracer administration using an Inveon microPET scanner (Siemens Medical Solutions, Erlangen, Germany). Vendor software (ASI Pro 5.2.4.0) was used to detect regions of interest using decay-corrected whole-body coronal images.

### Histology and immunohistochemistry

Tumour tissues fixed in paraffin blocks were cut into 8 µm sections and were stained with hematoxylin and eosin (Beyotime Institute of Biotechnology, Jiangsu, China). Sections were incubated (4ºC) with an anti-Ki67 antibody (Cat. #: ab15580, Abcam, Shanghai, China), followed by incubation with a horseradish peroxidase-conjugated secondary antibody. Immunohistochemical staining was conducted with a DAB substrate kit (Shanghai Bio-Platform Technology Company, Shanghai, China) and light microscopy (Olympus; Tokyo, Japan).

### TUNEL assay

TUNEL assay was used to detect cell death in paraffin-embedded tumour sections. Briefly, sections were placed on slides and stained with the TUNEL assay kit (Beyotime Institute of Biotechnology, Jiangsu, China), as described previously 51; nuclei were counter-stained with hematoxylin. Images were analysed using a KF-PRO-120 slide scanner (Konfoong Bioinformation Tech, Ningbo, China).

### Statistics

Data are presented as mean ± standard deviation (SD) with significance defined as p< 0.05. Observers were blinded in *in vivo* studies. Statistical analysis was conducted using one-way ANOVA and Dunnett's post-hoc test to compare multiple independent groups in GraphPad Prism 7.0.

## Results

### Lapatinib decreased the viability of UM cells

**Figure 1:**
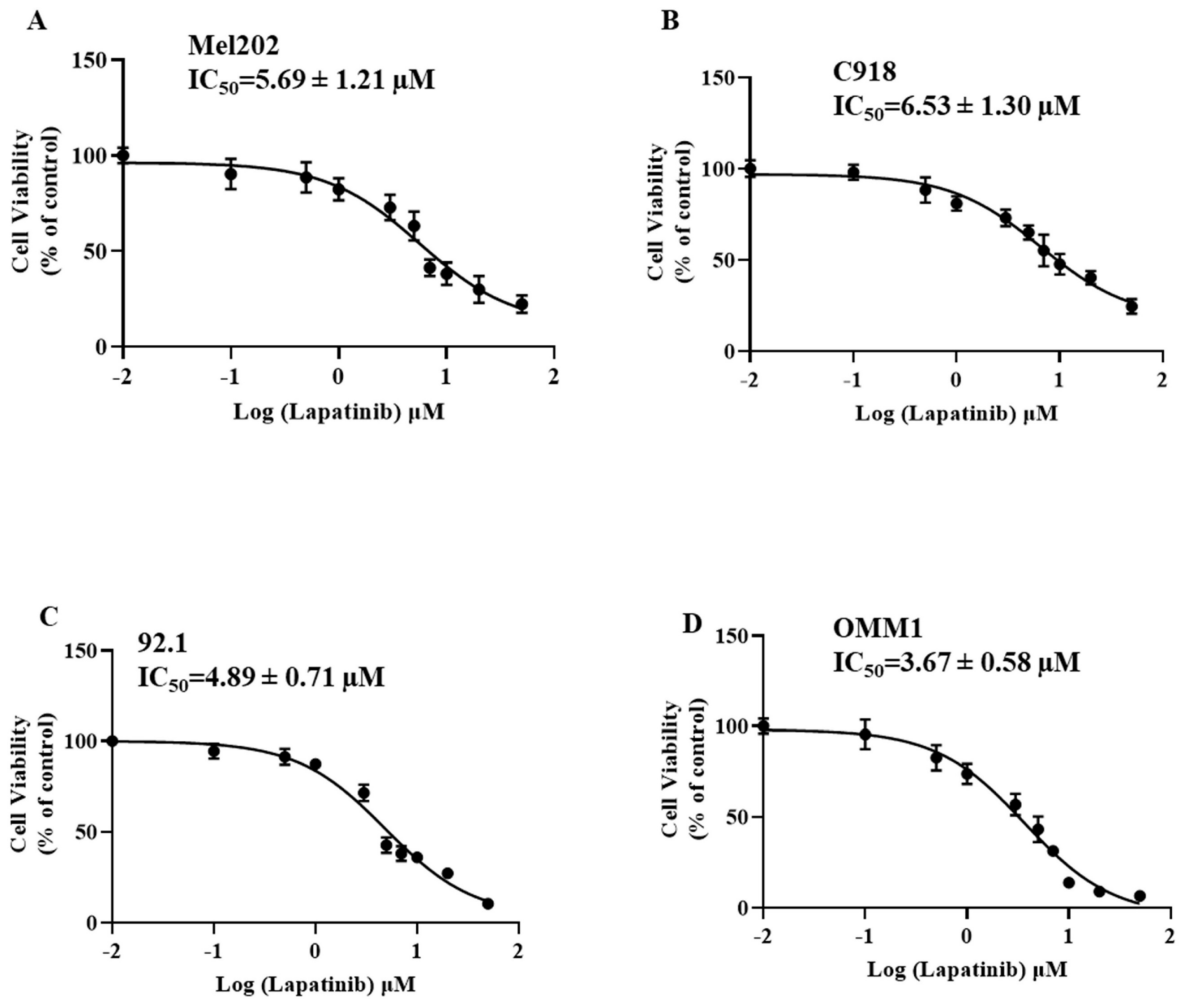
Lapatinib decreases the viability of UM cell lines. Mel202 (A), C918 (B), 92.1 (C) and OMM1 (D) cells were treated with lapatinib (0-50 µM) at 37°C for 24 h. Cell viability was assessed in MTT reduction assays. IC_50_s of lapatinib in UM cell lines were estimated by non-linear regression (GraphPad Prism 7.0; San Diego, CA).

The anti-UM activity of lapatinib were evaluated in C918, 92.1 and Mel202 cells that were derived from primary UM tumours and in OMM-1 cells that were isolated from a subcutaneous metastasis. All four cell lines were treated with lapatinib over the concentration range of 0 to 50 µM. Cell viability was then estimated using MTT reduction assays. As shown in Fig. 1, the IC_50_ values of lapatinib ranged from 3.67 µM to 6.53 µM across the four UM cell lines. It is noteworthy that the above-mentioned cell lines have BAP1 mutations. Therefore, we also investigated the effect of lapatinib in OCM-1 and MP46 cells in which BAP1 was mutated or absent. Similar IC_50_s were observed in these two cell lines (Suppl Fig. 1), which suggested that the effect of lapatinib is independent of BAP1 status.

### Lapatinib induced apoptosis and cell cycle arrest in UM cell lines

**Figure 2:**
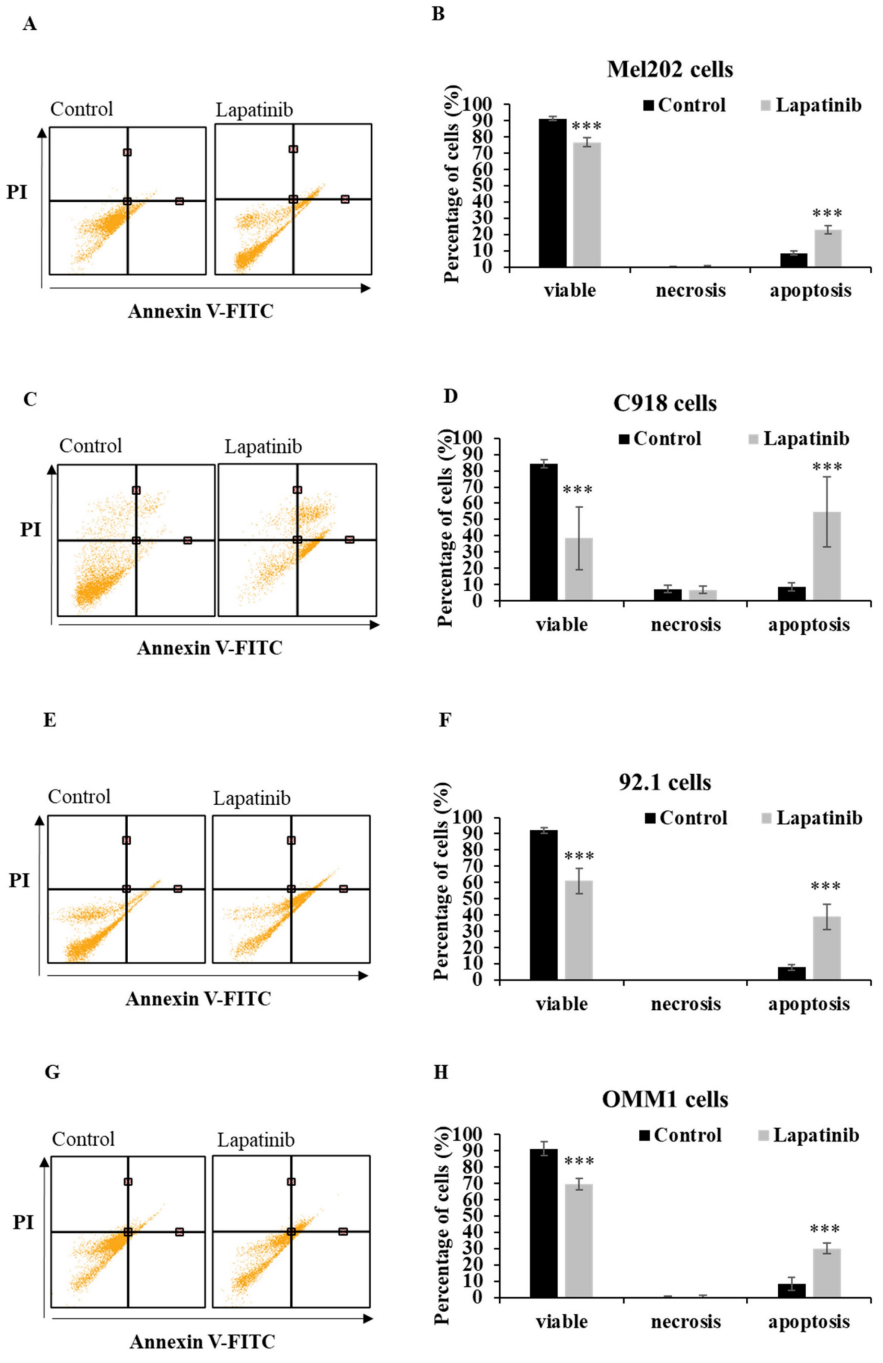
Lapatinib activates apoptosis in UM cell lines. Mel202 (A & B), C918 (C & D), 92.1 (E & F) and OMM1 (G & H) cells were treated with lapatinib (5 µM) at 37°C for 24 h, stained with Annexin V-FITC/PI and subjected to flow cytometry. Representative cell death profiles are shown for Mel202 (A), C918 (C), 92.1 (E) and OMM1 (G) cells. The percentages of viable, necrotic and apoptotic cells are indicated as mean ± SD for Mel202 (B), C918 (D), 92.1 (F) and OMM1 (H) cells. Experiments were performed on 3 independent occasions and each experiment included three repeats. Control treatments consisted of vehicle (DMSO) alone. ***p < 0.001 *vs.* control by One-way ANOVA and Dunnett's post-hoc test.

**Figure 3:**
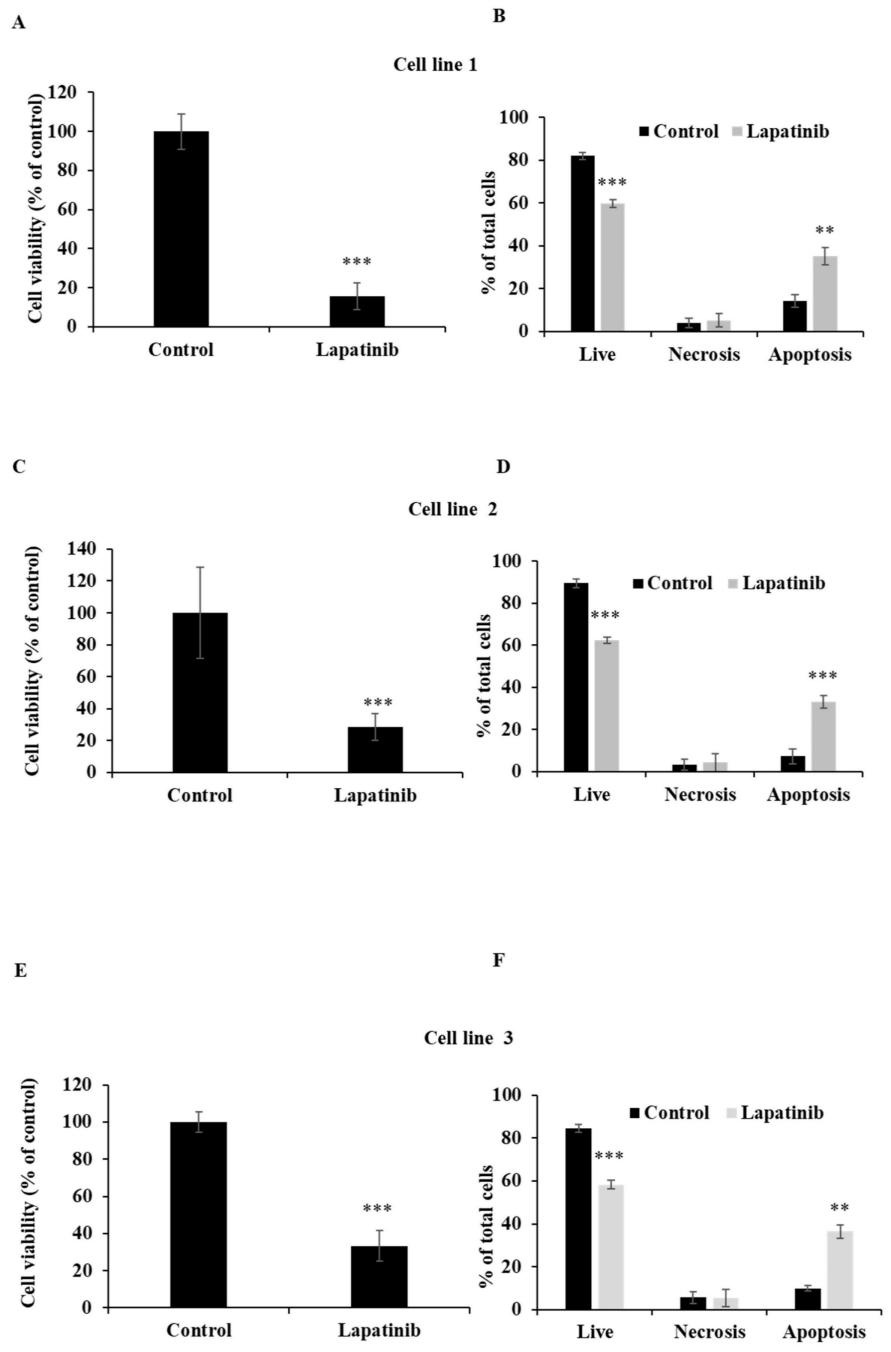
Lapatinib decreases viability and activates apoptosis in primary UM-tumour derived cell lines. UM tumour-derived cell lines were treated with lapatinib 5 µM for 24 h at 37°C. The viability of each primary cell line was assessed using MTT reduction (A, C and E). After treatment, cells were stained with Annexin V-FITC/PI and subjected to flow cytometry. The percentages of viable, necrotic and apoptotic cells are indicated as mean ± SD for each primary cell line in B, D and F. Experiments were performed on 3 independent occasions and each experiment included three repeats; control treatments consisted of vehicle (DMSO) alone. **p < 0.01; ***p < 0.001 vs. control by One-way ANOVA and Dunnett's post-hoc test.

The capacity of lapatinib to promote UM cell death was evaluated using Annexin-V/PI staining and flow cytometry. Apoptosis was found to be the principal cell death mechanism in all four UM cell lines after treatment with lapatinib (5 µM, 24 h; Fig. 2). Thus, lapatinib increased the proportion of apoptotic cells to 2.73-6.40-fold compared to control (p<0.001, Fig. 2B, 2D, 2F, 2H). In accord with these findings, lapatinib (5 µM) also decreased viability and activated apoptosis in three tumour-derived cell lines from UM patients (Fig. 3).

**Figure 4:**
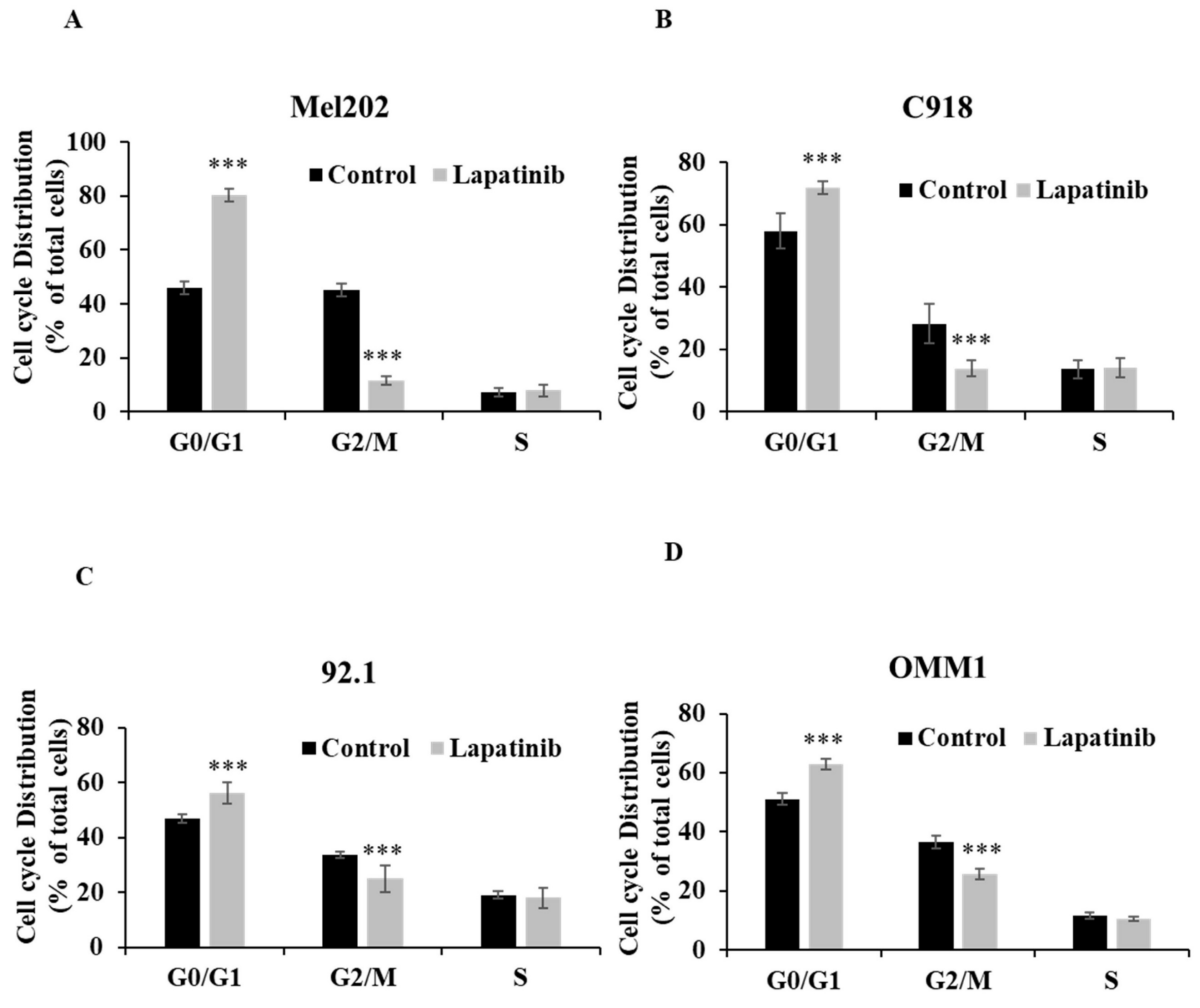
Lapatinib induces cell cycle arrest in UM cell lines. Mel202 (A), C918 (B), 92.1 (C) and OMM1 (D) cells were treated with lapatinib (5 µM) for 24 h at 37°C. Cells were stained with PI and subjected to flow cytometry. The percentages of cells in G_0_/G_1_, G_2_/M and S phases are shown as mean ± SD. Experiments were performed on 3 independent occasions and each experiment included three repeats; control treatments consisted of vehicle (DMSO) alone. ***p < 0.001 vs. control by One-way ANOVA and Dunnett's post-hoc test.

The cell cycle arrest assay is to indicate the influence of treatment on cell cycle progression, which is relevant to cell death. The inhibitory effect (IC_50_) is an overall outcome from lapatinib-induced cell death and cell cycle arrest, so it is a functional read-out of the composite anti-cancer effects of lapatinib in UM cell lines*.* To further evaluate the impact of lapatinib on viability, UM cells were stained with PI and subjected to cell cycle analysis by flow cytometry. The proportion of cells in G_0_/G_1_ phase was increased by lapatinib (5 µM, 24 h; P<0.001), while the proportion of cells in G_2_/M phase was decreased (P<0.001) and cells in S phase were unchanged (Fig. 4). Taken together, these findings indicate that lapatinib is highly effective in inducing apoptosis and cell cycle arrest in UM cell lines.

### Lapatinib modulates STAT1 and apoptotic signaling in UM cells

**Figure 5:**
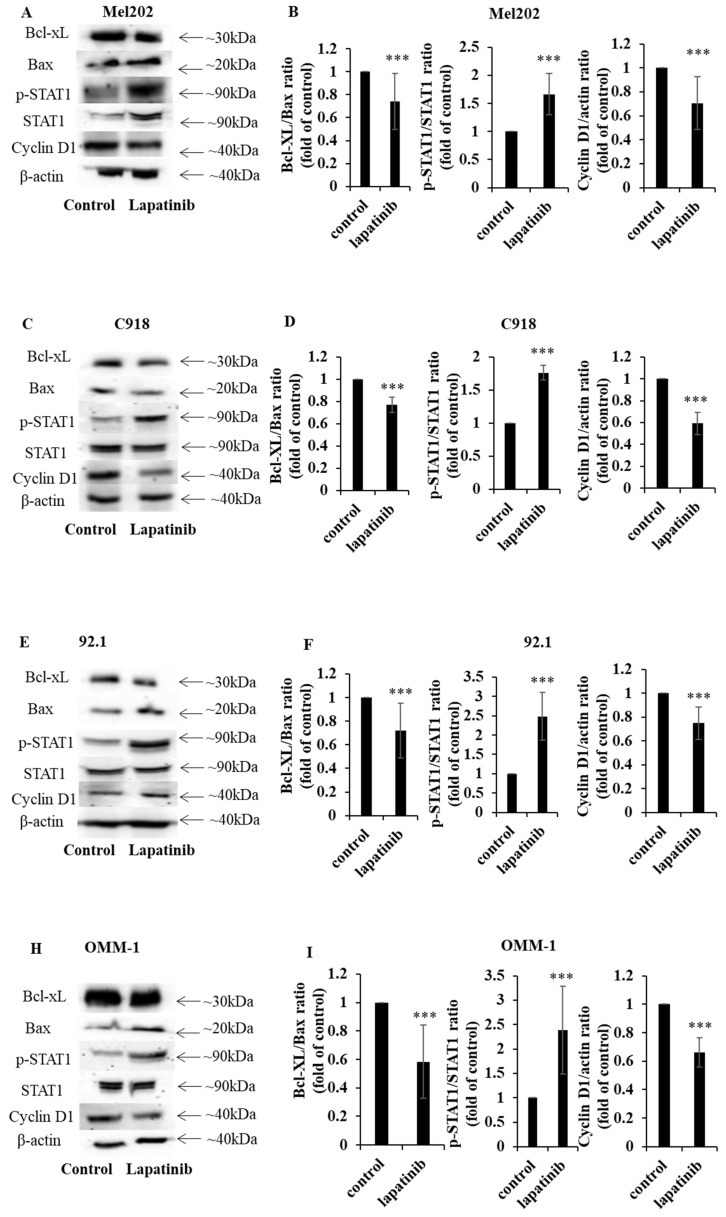
The lapatinib-mediated activation of apoptosis is associated with the modulation of STAT1, Bcl-XL and cyclin D1 expression in UM cell lines. Expression of Bcl-XL, BAX, p-STAT1, STAT1 and cyclin D1 was determined by Western blotting with β-actin as the loading control. Cells were treated with lapatinib (5 µM) at 37°C for 24 h, then harvested, lysed, denatured and subjected to sodium dodecylsulfate-polyacrylamide gel electrophoresis. Representative images of proteins of interest are shown for Mel202 (A), 92.1 (C), C918 (E) and OMM-1 (H) cells. Densitometry analysis for protein quantification was conducted using Image J. Bcl-XL:BAX and p-STAT1: STAT1 expression ratios as well as the expression of cyclin D1 relative to β-actin are shown for Mel202 (B), C918 (D), 92.1 (F) and OMM1 (I) cells as fold of control (mean ± SD); control treatments consisted of vehicle (DMSO) alone. Experiments were performed on three separate occasions. ***p < 0.001 vs. control by unpaired t-test.

STAT1 is an important regulator of apoptosis 52, 53. In the present study, the capacity of lapatinib to modulate the expression of STAT1 and its downstream signaling was examined. Treatment with lapatinib substantially increased STAT1 expression to 1.4 - 4.9-fold compared to control across all four UM cell lines (Fig. 5A, 5C, 5D, 5F, 5G, 5I, 5J, 5L). Further, lapatinib decreased the expression of the anti-apoptotic Bcl-XL and increased the pro-apoptotic BAX in all four UM cell lines (Fig. 5). Consistent with the activation of apoptosis Bcl-XL:BAX ratios were markedly decreased by lapatinib (5 µM, 24 h), as shown in Fig. 5B, 5E, 5H and 5K.

Because lapatinib induced cell cycle arrest in UM cell lines (Fig. 4) we assessed the expression of cyclin D1- a key cell cycle mediator that is also downstream from STAT1. Treatment with lapatinib (5 µM, 24 h) decreased cyclin D1 expression in UM cells to 0.24-0.47 fold compared to control (Fig. 5C, 5F, 5I and 5L).

In summary, lapatinib induced cell death was associated with dysregulated expression of STAT1 and its downstream targets cyclin D1, BAX and Bcl-XL.

### Lapatinib inhibited UM cell migration, invasion and suppressed reproductive growth

**Figure 6:**
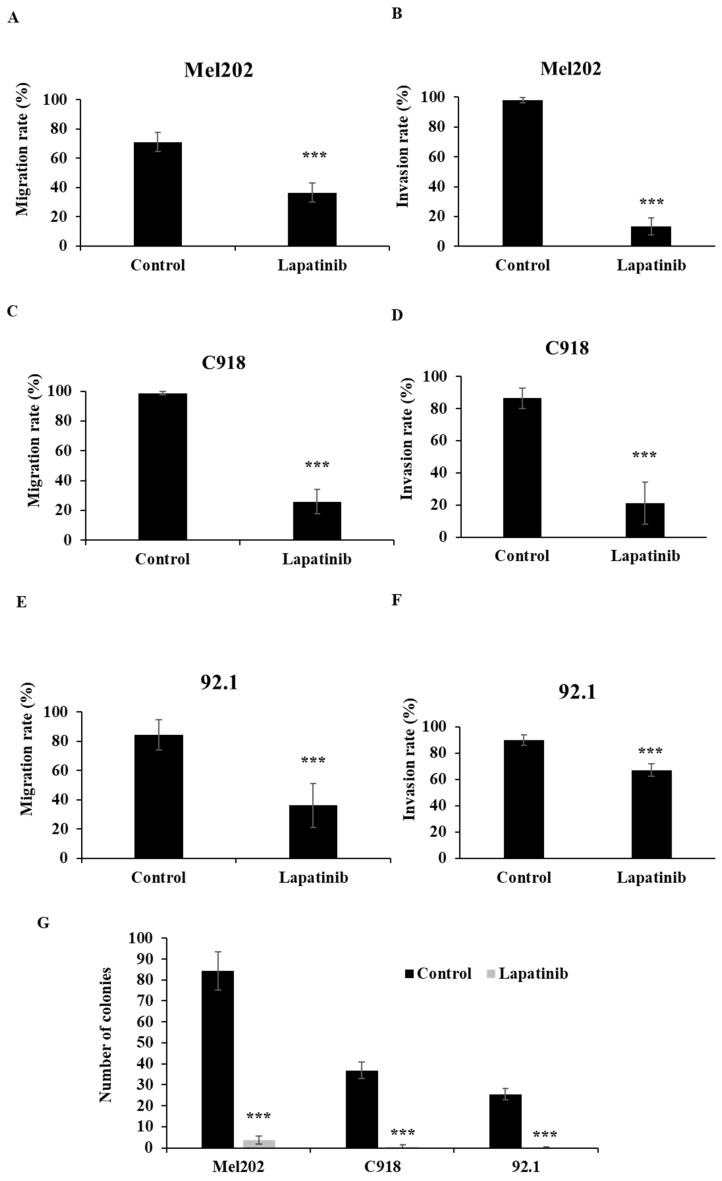
Lapatinib is anti-metastatic in primary tumour-derived UM cell lines. The anti-migratory actions of lapatinib were assessed in scratch-wound assays. UM cell lines were treated with lapatinib (5 µM) at 37°C for 24 h. Cell images were captured at 0 and 24 h. The rate of cell migration was estimated as the means of each repeat and are indicated as percentage of control (means ± SD) for Mel202 (A), C918 (C) and 92.1 (E). Cell invasion upon lapatinib treatment was studied in Matrigel invasion assays. UM cell lines were treated with lapatinib (5 µM) at 37°C for 24 h to 72 hr. Cell images were captured at 0 and 24 to 72 h. The rate of cell invasion was estimated as the means of each repeat and are indicated as percentage of control (means ± SD) for Mel202 (B), C918 (D) and 92.1 (F). Reproductive cell growth after lapatinib treatment was evaluated in colony formation assays. (G) Colony number is indicated as the percentage of control (mean ± SD). Experiments were performed on 3 independent occasions and each experiment included four repeats; control treatments consisted of vehicle (DMSO) alone. ***p < 0.001 vs. control by One-way ANOVA and Dunnett's post-hoc test

The migration, invasion and colony formation assay (tumor reproductive growth analysis) are well established methods to evaluate the anti-metastatic effect of drugs. The impact of lapatinib on UM cell migration was examined in scratch-wound healing assays. As shown in Fig 6A, 6C and 6E, lapatinib decreased rates of migration in the Mel202, C918 and 92.1 cell lines that were derived from primary UM tumours to 26% - 36% of control (P < 0.001).

Lapatinib also potently inhibited the invasion of UM cells in the Matrigel invasion assay. As shown in Fig. 6B, 6D and 6F, treatment with lapatinib reduced cell invasion rates of Mel202, C918 and 92.1 cell lines to 13% - 67% of control (P<0.001).

Colony formation assays were also performed to assess reproductive cell growth upon lapatinib treatment (5 µM, 24 h). As shown in Fig. 6G, lapatinib significantly decreased the number of viable colonies post treatment in all three cell lines (P<0.001).

These findings suggest that lapatinib has anti-metastatic actions in UM.

### Lapatinib exerts its anti-UM activity by inhibiting HER2 signalling

Lapatinib is an established inhibitor of HER2 and is used clinically in the treatment of HER2-positive cancers, including breast cancers that are resistant to the first-choice agent trastuzumab 54-56. Unlike other ErbB receptor isoforms, it has been found previously that HER2 is uniformly expressed in UM cells 37, 57, 58.

**Figure 7:**
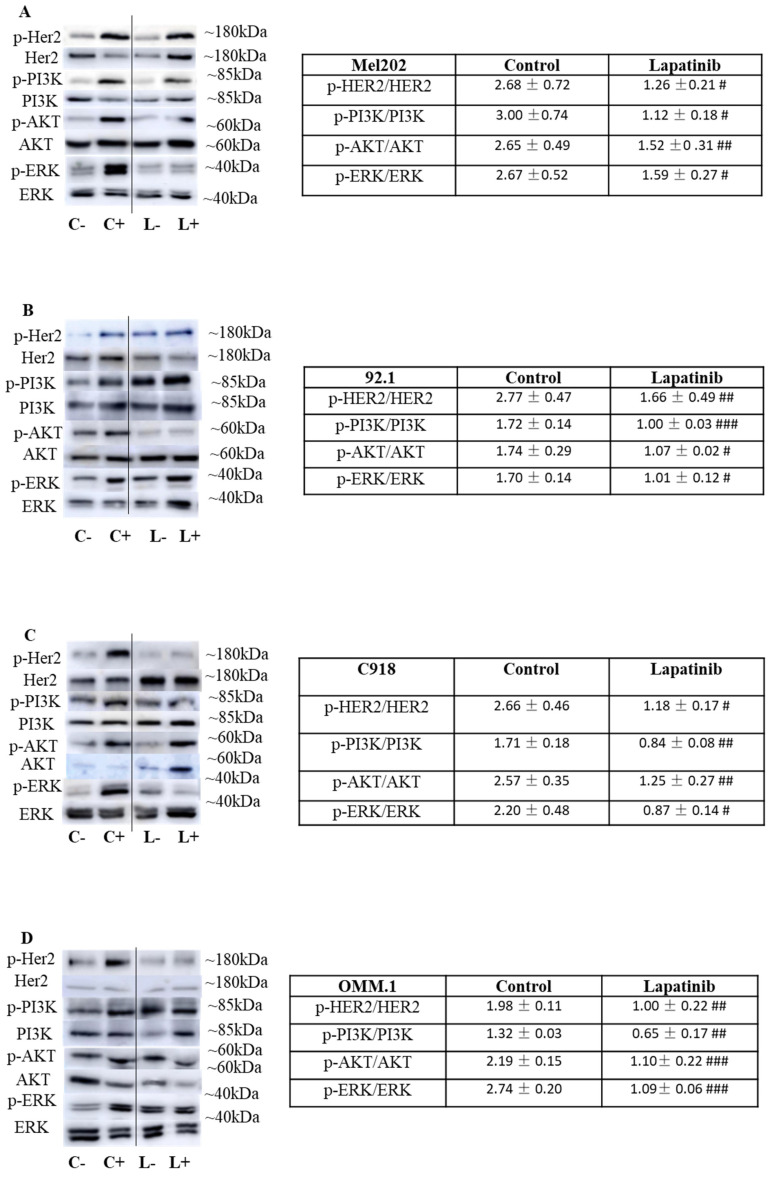
The lapatinib-mediated activation of cell death is associated with inhibition of HER2 and its downstream signaling cascades in UM cell lines. In each experiment, four sets of cells were cultured in serum-free medium for 24 h. Two of the four sets of cells were then treated for 10 min with medium containing 20% FBS while the other two sets of cells remained serum-free. In the next step, one each of the sets of serum-treated and serum-free cells was treated with lapatinib (5 µM) at 37°C for 1 h, while the others were treated with vehicle alone (DMSO), and lysates were prepared. This produced a four-way design that evaluated the effect of serum addition and lapatinib addition on the signalling pathways (Key: C-: vehicle control without serum stimulation; C+: vehicle control with serum stimulation; L-: lapatinib treatment without serum stimulation; L+: lapatinib treatment with serum stimulation). Expression of HER2, AKT, ERK and PI3K and their phosphorylated isoforms was evaluated by Western blotting and densitometry analysis. Representative images of p-HER2, HER2, p-AKT, AKT, p-PI3K, PI3K, p-ERK and ERK are shown for Mel202 (A), 92.1 (B), C918 (C) and OMM-1 (D) cells. Densitometry analysis was conducted using ImageJ and the ratios (L+/L-) and (C+/C-) were calculated for the effects on lapatinib and DMSO respectively on the expression of phosphorylated and total forms of the proteins. These data, as fold of corresponding control (mean ± SD; no serum stimulation), are shown in the Tables to the right of panels A-D. Experiments were repeated on three occasions. #p < 0.05; ##p < 0.01; ###p < 0.001 vs. control by Two-way ANOVA.

We assessed the impact of lapatinib on the expression of HER2 and its phosphorylated isoform in the four UM cell lines. In these experiments, cells were initially cultured in serum-free medium and were then treated with 20% FBS for 10 min immediately prior to treatment with lapatinib (L+; Fig. 7) or vehicle (C+; Fig. 7). This rapidly activated HER2 phosphorylation that was attenuated by lapatinib (Fig. 7; compare the values for lapatinib and control in the Tables at right). Important downstream targets of HER2 include ERK, PI3K and AKT. Inclusion of lapatinib also prevented the activation of these pathways after serum addition (Fig. 7; compare L+ *versus* L- relative to C+ *versus* C-). Noteworthy, the comparison of protein expression in Fig. 7 is the ratio of phosphorylated and total forms of HER2, PI3K, AKT and ERK in each treatment. The samples for estimation of each pair of proteins were obtained in parallel analyses from the same experimental treatment. Thus, loading controls in each panel are not required.

To our knowledge, the expression of HER2 has not been reported in population or similar studies of UM patients. Based on our own observation, the expression of HER2 varies widely across multiple UM cell lines. We also qualitatively confirmed the expression of HER2 protein in the three primary tumor-derived cell lines used in the present study (Suppl Fig. 2). Importantly, we also explored the Cancer Genome Atlas Program (TCGA) database (https://portal.gdc.cancer.gov/). In a cohort of 80 UM patients, ErbB2 (HER2) was expressed in all tumor samples and its expression was significantly higher than EGFR and ErbB4 (p<0.001) (Suppl Fig. 3). In addition, we demonstrated the effectiveness of HER2 inhibitors, especially afatinib, lapatinib and neratinib, in UM cells in the present study and in our previous paper 37. In the literature, Forsberg et. al has also investigated HER2 as a possible target and reported HER2 to be expressed in UM 57. Overall, these findings indicate that lapatinib inhibits HER2 and its downstream signaling and suggests that these may be early events in its anti-UM activity.

### Lapatinib has potent anti-tumour activity in a UM xenograft mouse model

**Figure 8:**
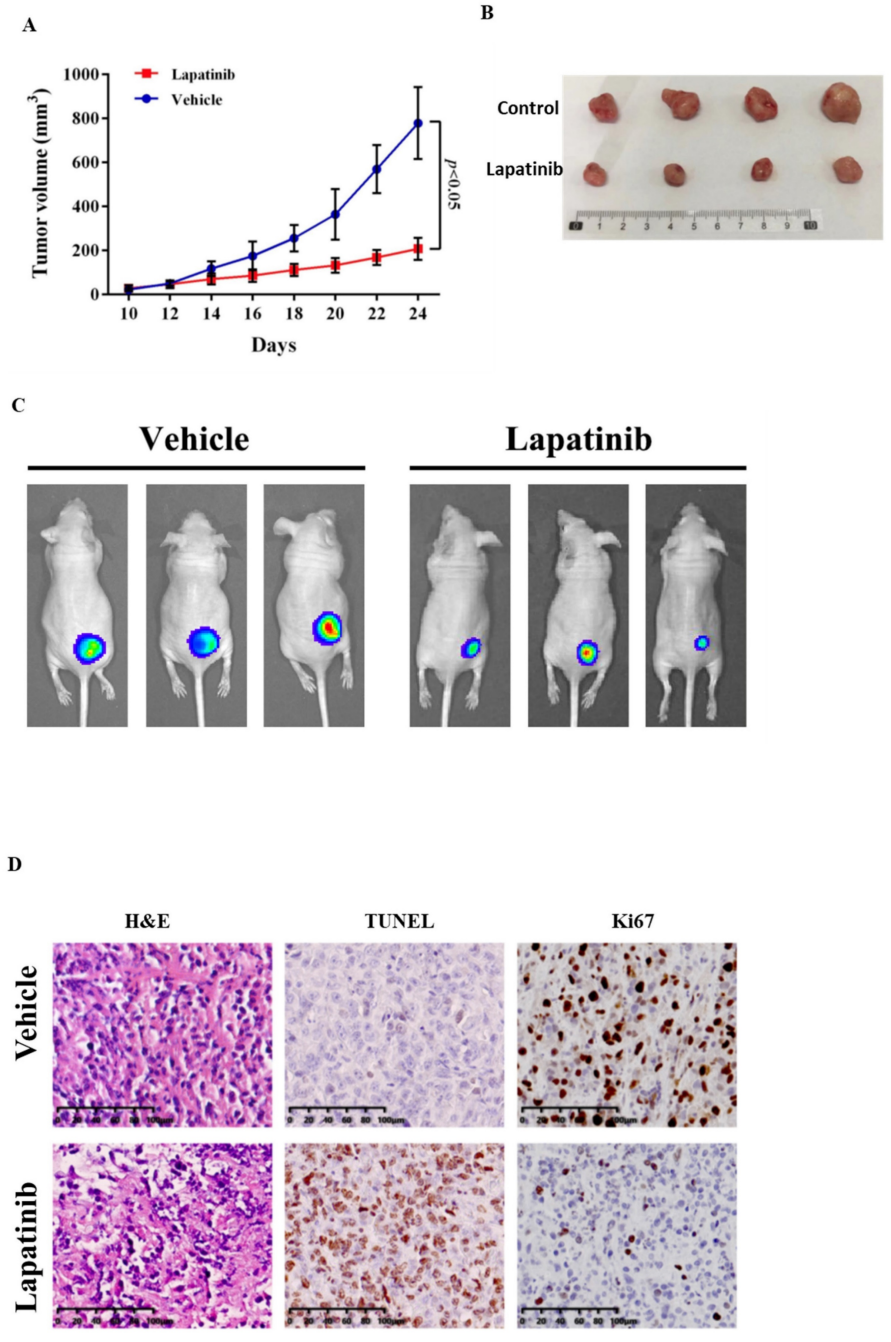
Lapatinib inhibited tumour growth in UM xenografted mice. BALB/c nude mice were inoculated with C918 cells. After 14 d, mice received either lapatinib (25 mg/kg per day, n = 10) or vehicle (n = 12) on day 10 by intraperitoneal injection; treatments were continued for a further 14 d. Tumour volumes and body weight of mice were measured every 2 d. At the end of the experiment, mice were either sacrificed to harvest tumour samples or were subjected to whole body PET scan (n = 5 for lapatinib and 6 for vehicle). Tumour size *vs* treatment time is indicated in (A) and representative tumour images at the end of experiment are shown in (B). Data are presented as tumour volumes at each time point (mean ± SD; n = 5 for lapatinib and 6 for vehicle); p < 0.05 vs. control by unpaired t-test. Representative PET scans are shown in (C). Harvested tumours were embedded in paraffin and sections were prepared for staining. Representative images of hematoxylin and eosin (H&E) staining of tumour sections are shown in the panels at left, TUNEL staining is indicated in the central panels and Ki67 staining is shown in the right panels (D).

The anti-UM activity of lapatinib was examined further in a xenograft model 51. Lapatinib (25mg/kg for 14 d) was administered to nude mice that carried UM cell xenografts: tumour growth was suppressed (Fig. 8A and 8B). From PET scan analysis, the final tumour sizes in the lapatinib-treated mice were smaller than those in controls (Fig. 8C).

Confirmatory immunohistochemical staining was undertaken in tumour samples that were collected at the end of the experimental treatments. From H&E staining the tumour architecture was improved by lapatinib treatment (Fig. 8D). Staining of the cell proliferation marker Ki67 was decreased by lapatinib and apoptosis as reflected by TUNEL staining, was increased (Fig. 8D).

Overall, these data indicate that lapatinib inhibits tumour growth, suppresses cell proliferation, and activates tumour cell apoptosis *in vivo* in mice carrying UM-cell xenografts.

## Discussion

ErbB receptors regulate cellular homeostasis. Dysregulation of the receptors leads to impairment of proliferative and pro-survival mechanisms in cells and may contribute to disease progression 59-61. Intracellular signaling cascades downstream from ErbB receptors are regulated by phosphorylation events that are mediated by kinase intermediates. The development of small molecule inhibitors of ErbB receptor-linked kinases has revolutionised the treatment of a number of cancers 62. The ErbB receptor member EGFR was initially suggested to be a potential drug target in UM. However, EGFR inhibitors like gefitinib have been disappointing in clinical trials that have been conducted in UM patients 26, 38, 40, 63. Despite these outcomes, small molecules that target other members of the ErbB family have not been widely considered as alternative agents for use in patients with UM.

We found previously that the EGFR, HER2 and HER4 inhibitor afatinib, was an effective anti-cancer and anti-metastatic agent in UM 37. EGFR inhibition appears to be of limited value in UM 37, 39, 64. None of the UM cell lines included in the previous and current study express EGFR; and two of the four UM cell lines do not express HER4 37. Therefore, these receptors are unlikely to be the primary targets for afatinib. In contrast, HER2 is expressed in UM tumours as well as the four UM cell lines included in this study (Fig. 7 and Supplementary Fig. 2) 37, 57, 65. Moreover, we also explored the Cancer Genome Atlas Program (TCGA) database (https://portal.gdc.cancer.gov/). In a cohort of 80 UM patients, HER2 was expressed in all tumor samples and its expression was significantly higher than EGFR and HER4 (p<0.001) (Suppl Fig. 3). Thus, it is now appropriate to evaluate in greater detail the potential clinical value of HER2 targeting in the treatment of UM.

Lapatinib is a high affinity HER2 inhibitor (Table 1) 66, and is currently approved in combination with cytotoxic agents such as capecitabine for HER2-positive breast cancers 67-69. Lapatinib is a reversible inhibitor of the kinase binding site of HER2, and blocks downstream proliferative and pro-survival signaling 67. Lapatinib has advantages of receptor targeting specificity over afatinib. Afatinib is 28-fold more potent against EGFR than HER2, and is also effective against common mutant EGFRs, whereas the relative activity of lapatinib against HER2 is greater (Table 1) 70. Thus, off-target effects at EGFR in multiple tissues are expected to be less likely with lapatinib. The previous studies have demonstrated the cytotoxic effect of lapatinib in melanoma cell lines 71, 72, but its influence on UM cell lines remains unclear. In the present study, we investigated the anti-cancer and anti-metastatic actions of lapatinib in a range of UM models for the first time. Lapatinib decreased UM cell viability by inhibiting cell proliferation and by promoting apoptosis and cell cycle arrest. Lapatinib also decreased tumorigenesis *in vivo* in mice that carried UM cell xenografts.

Afatinib and lapatinib have different efficacies against other cancer types 73. Compared to afatinib 37, lapatinib was more effective in inhibiting UM cell migration and reproductive cell growth, which suggests that it may have utility in the suppression of UM metastasis. In contrast, afatinib was slightly more effective in the induction of cell apoptosis and cell cycle arrest (Table 1). Taken together, these findings suggest that afatinib may be considered for the treatment of primary UM tumours. And lapatinib may be used as an adjuvant therapy in the prevention of UM metastasis following the non-pharmacological treatment of primary UM tumours or be applied to UM patients together with agents like tebentafusp (an FDA approved immunotherapeutic drug of UM) in the future.

HER2 is not activated by ligand binding but is instead a signal transducer that heterodimerises with other ErbB receptors that are ligand activated 37, 74. Because EGFR was not detected in any of the four UM cell lines tested in this study 37, and HER4 is only expressed in two of the four cell lines (data not shown), these receptors are unlikely to be required for the anti-UM activity of lapatinib or afatinib.

HER2 is an important driver of tumourigenesis in several cancers, including HER2-positive breast cancers where its expression is amplified 75. HER2 overexpression or activation in breast cancer is often accompanied by poor prognosis due to more aggressive and invasive behaviour 76, 77. HER2 expression was inversely correlated with outcomes from breast cancer treatment 78-80. HER2 activation is also associated with increased tumour size and invasiveness 81, 82. In a large study (n=1,012), ~37% of patients with HER2 positive breast cancer reportedly had brain metastases 83.

HER2 is linked to the activation of multiple downstream signalling pathways that regulate tumorigenesis, including STAT1-regulated cascades 84-86. STAT1 regulates an array of complex cellular processes, notably in tumour cells and in the immune system, as an anti-proliferative and pro-apoptotic gene 87. STAT1 regulates cell cycle progression and the inhibition of HER2 was found to increase STAT1 expression and promote cell cycle arrest by downregulation of cyclin D1 84, 88 which suppresses tumorigenesis 89, 90. STAT1 physically interacts with cyclin D and forms a complex with G_1_ CDK to mediate IFN-γ-dependent G_1_ cell cycle arrest 84. STAT1 also regulates the transcription of Bcl-2 genes that modulate apoptosis. Thus, the activation of STAT1 upregulates pro-apoptotic BAX and downregulates the anti-apoptotic Bcl-2 and Bcl-XL 91. In the present study, lapatinib promoted cell cycle arrest and apoptosis by decreasing the expression of cyclin D1 and the anti-apoptotic Bcl-XL.

HER2 also regulates the AKT, ERK and PI3K-linked signaling pathways that modulate cell proliferation, migration, and death 92 and that contribute to tumorigenesis in multiple cancer types 93, 94. The overarching consensus is that the activation of HER2-AKT/ERK/PI3K cascades increases cell proliferation, survival and migration 95-97. The present findings that lapatinib impairs PI3K, Akt and ERK signalling downstream from HER2 are consistent with its antiproliferative and antimigratory actions in UM cells.

Lapatinib has additional advantages that could facilitate its clinical translation. The anti-cancer activity of lapatinib due to HER2 inhibition has been established in studies of HER2-positive breast cancer, including advanced metastatic disease 98-100. Lapatinib is currently administered orally in a once daily dosage regimen (dose range 100 to 1,500 mg per day) and produces C_min_ values in the range 0.29-0.77 µM and C_max_ values in the range 0.70-5.63 µM 101, 102. These plasma concentrations likely fall within the range of those required for effective anti-UM activity (Fig. 1). Lapatinib also crosses the blood brain barrier, because it has been shown that brain metastases were decreased to 50%-53% of control in xenografted mice with metastatic breast cancer 98. Noteworthy, lapatinib is also under clinical investigations for several other solid tumours with high EGFR and/or HER2 expression 103.

**Figure 9:**
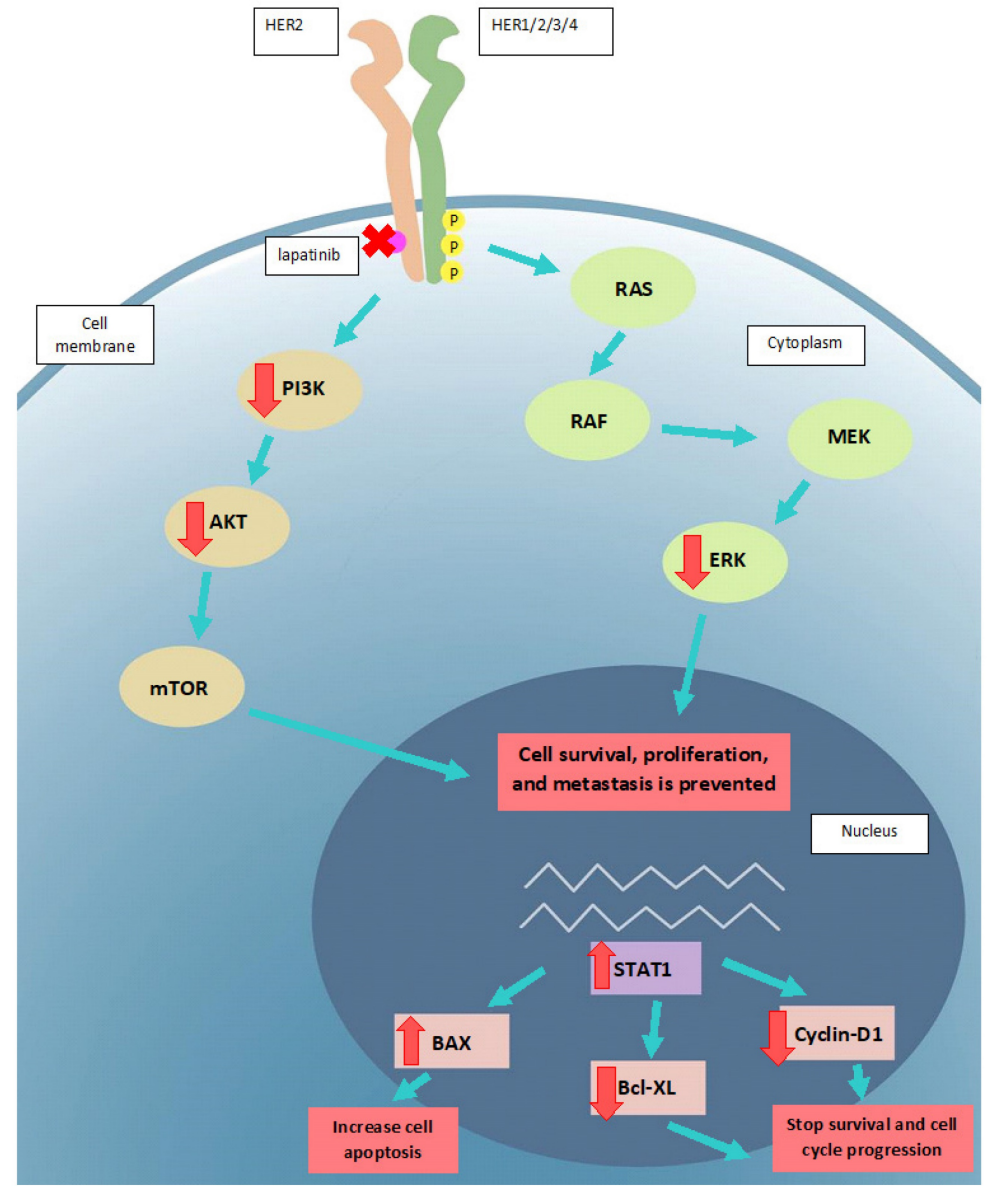
The proposed mode of action of lapatinib in UM cell lines. Lapatinib inhibits HER2 and its downstream signaling along PI3K/AKT and Ras/MEK/ERK pathways. UM apoptosis is activated by upregulation of BAX and STAT1 as well as a downregulation of Bcl-XL and cyclin D1. Key: intracellular p, phosphorylated residues in receptors; AKT: Protein kinase B; BAX: Bcl-2-associated X Protein; Bcl-XL: B-cell lymphoma-extra large; ERK: extracellular-signal-regulated kinase; MEK: Mitogen-activated protein kinase kinase; mTOR: mammalian target of rapamycin; RAS: RAS viral oncogene homolog; PI3K: Phosphatidyinositol 3-Kinase; RAF: rapidly activated fibrosarcoma; STAT1, signal transducer and activator of transcription-1.

The present study found that lapatinib decreased tumour cell migration, invasion and reproductive growth, which suggests that the drug may be developed as an adjuvant therapy in the prevention of UM metastases. The finding that the anti-cancer actions of lapatinib are consistent with inhibition of HER2 and its downstream targets supports the potential utility of lapatinib in UM (Fig. 9). And this finding is aligned with the report of Ma *et al*. that UM patients with higher risk and lower overall survival rates are more susceptible to drugs including lapartinib 104. Clinical trials to test this directly in UM patients may now be warranted.

It has been recognised that the usual systemic administration route may not be optimally effective in delivering lapatinib to UM patients. However, with the development of drug delivery technology, novel drug carriers may be used to facilitate the localised delivery of agents. For example, nanoparticles can enhance drug permeability, increase stability and control release rate; these are ideal carriers for targeted drug delivery. Specific nanoparticles, including albumin, chitosan and other natural polymer nanoparticles, have been shown to effectively penetrate the eye allowing for improved ocular drug delivery 105. Future studies are warranted to investigate the suitability of this drug delivery route for lapatinib in the treatment of UM; however, this is beyond the scope of the current study.

This principal findings from the present study are that lapatinib is a potential candidate for the treatment of UM, based on its anti-cancer and anti-metastatic activities in *in vitro*, *ex vivo* and *in vivo* models. Importantly, the present study supports the assertion that HER2 is a promising therapeutic target in UM. Taken together, lapatinib is a model HER2 inhibitor that is already approved for the treatment of HER2-positive breast cancer that could now be evaluated further in clinical trials in UM patients.

## Supplementary Material

Supplementary figures.

## Figures and Tables

**Table 1:**
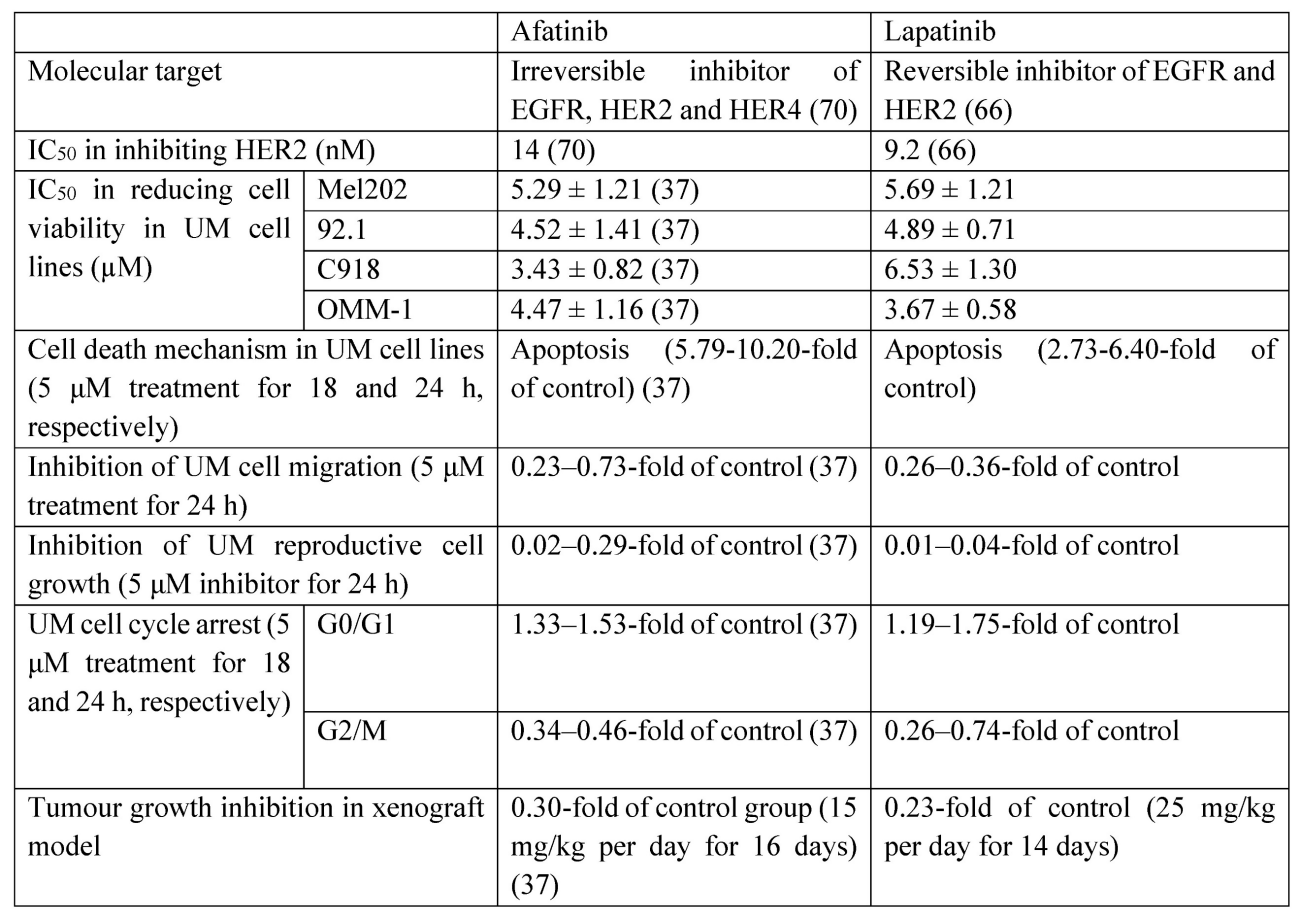
The comparison of the anti-UM effects of afatinib and lapatinib

## Abbreviations

AKT: Protein kinase B
BAX: Bcl-2-associated X Protein
Bcl-XL: B-cell lymphoma-extra large
BRAF: v-raf murine sarcoma viral oncogene homolog B1
DMEM: Dulbecco's Modified Eagle Medium
EGFR: Epidermal Growth Factor Receptor
ERK: extracellular-signal-regulated kinase
FBS: Fetal Bovine Serum
GCT: Giant cell tumour
HER: Human Epidermal Growth Factor Receptor
ITS: Insulin-Transferrin-Selenium
JAK: janus kinase
KIT: tyrosine-protein kinase
MAPK: Mitogen-Activated Protein Kinase
MEK: Mitogen-activated protein kinase kinase
MTT: thiazolyl blue tetrazolium bromide
mTOR: mammalian target of rapamycin
NRAS: neuroblastoma RAS viral oncogene homolog
PBS: phosphate-buffered saline
PI: propidium iodide
PI3K: Phosphatidyinositol 3-Kinase
PKC: Protein kinase C
PLCγ: Phospholipase C Gamma
P/S: Penicillin-Streptomycin
RAS: Rat sarcoma virus
STAT: signal transducer and activator of transcription
UM: uveal melanoma
