# Supplementary material for: Lapatinib dysregulates HER2 signaling and impairs the viability of human uveal melanoma cells
Source: J Cancer. 2023 Oct 16;14(18):3477–95. doi: 10.7150/jca.88446 (PMC10647189; doi:10.7150/jca.88446)
Supplement: Supplementary file 1 — Supplementary figures. [file jcav14p3477s1.pdf]

Supplementary data

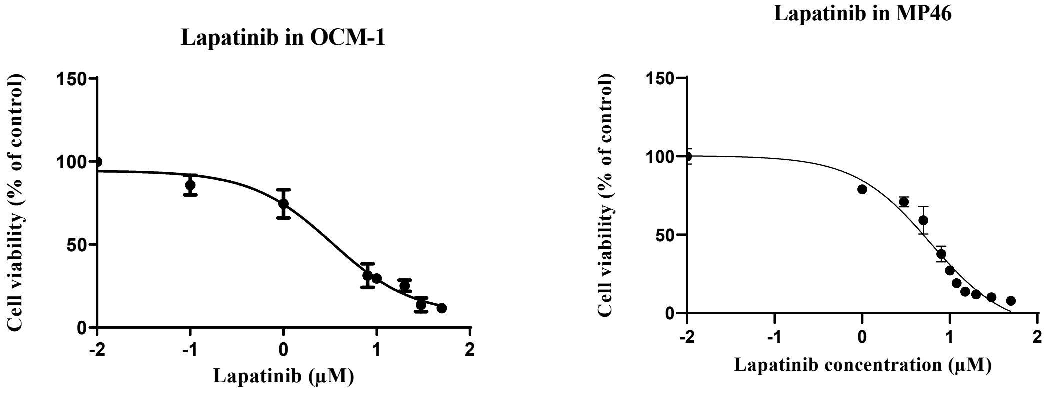

IC_{50}= 3.29 ± 0.87 μM IC_{50}=6.01 ± 1.52 μM

Supplementary Figure 1. Anti-cancer activity of lapatinib in OCM-1 and MP46 UM cell lines.

OCM-1 and MP46 cells were treated with lapatinib (0-50 μM) at 37°C for 24 h. Cell viability was assessed using MTT reduction assays. IC_{50}s of lapatinib in UM cell lines were estimated by non-linear regression (GraphPad Prism 7.0; San Diego, CA).

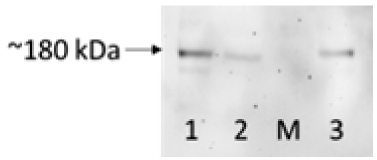

Supplementary Figure 2. Protein expression of Her2 in the patient tumor-derived UM primary cell lines.

The total cell lysate of the three patient tumor-derived UM primary cell lines was subject to western blot analysis. The blots were probed with anti-Her2 antibody.

Lanes: 1: cell line 1; 2: cell line 2; 3: cell line 3; M: protein marker.

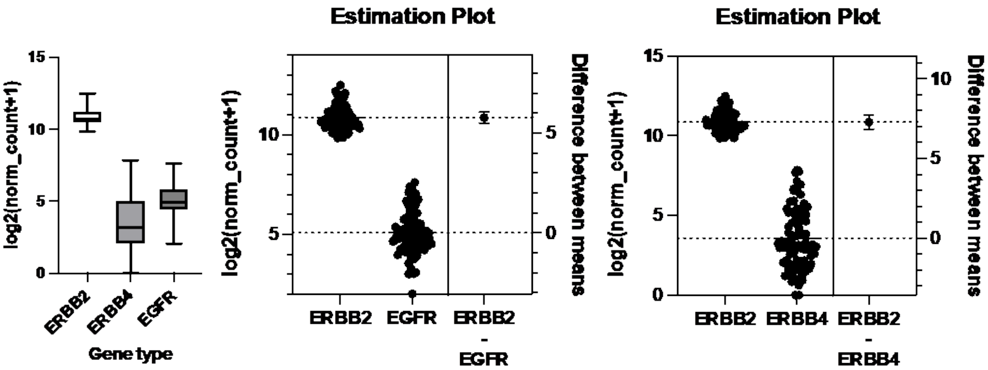

Supplementary Figure 3 The expression of HER2 (ERBB2), EGFR and HER4 (ERBB4) in UM from the TCGA database.

The expression of EGFR and HER4 was compared with that of HER2 via the unpaired t-test (the middle and right panels).

HER2 *vs* EGFR: $p<0.0001$, t=41.00, df=158, mean of HER2=10.87, mean of EGFR=5.089

HER2 *vs* HER4: $p<0.0001$, t=32.89, df=158, mean of HER2=10.87, mean of HER4=3.578
